# Atherectomy Followed by Drug-Coated Balloon Angioplasty Versus Surgery for Symptomatic Deep Femoral Artery Arteriosclerotic Disease

**DOI:** 10.1177/15266028241284443

**Published:** 2024-10-18

**Authors:** Giovanni Battista Torsello, Ryan Gouveia e Melo, Thomas Zeller, Tanja Böhme, Grigorios Korosoglou, Raphael Coscas, Konstantinos Stavroulakis, Dimitrios Kapetanios, Giovanni Federico Torsello, Bahaa Nasr

**Affiliations:** 1 Institute for Vascular Research, St. Franziskus Hospital, Münster, Germany; 2 Department of Vascular Surgery, Unidade Local de Saude Santa Maria (ULSSMM), Lisbon, Portugal; 3 Centro Cardiovascular da Universidade de Lisboa, Lisbon, Portugal; 4 Department of Cardiology and Angiology, University Heart Center Freiburg-Bad Krozingen, Bad Krozingen, Germany; 5 Departments of Cardiology, Vascular Medicine and Pneumology, GRN Academic Teaching Hospital Weinheim, Weinheim, Germany; 6 Department of Vascular Surgery, CHU Ambroise Paré, Hôpitaux de Paris (AP-HP), Boulogne-Billancourt Cedex, France; 7 Department of Vascular and Endovascular Surgery, Ludwig-Maximilian-University Hospital, Munich, Germany; 8 Institute of Radiology, University of Göttingen, Göttingen, Germany; 9 Department of Vascular and Endovascular Surgery, Univ Brest, CHU Brest, UMR 1101 LaTIM, Brest, France

**Keywords:** peripheral arterial disease, profunda atherectomy, profundaplasty, claudication, chronic limb threatening ischemia, drug-coated angioplasty

## Abstract

**Purpose:**

Limited data are available regarding endovascular therapy of arteriosclerotic lesions of the deep femoral artery (DFA). In this study, we compare the outcomes of atherectomy combined with drug-coated balloon (DCB) angioplasty and open repair of DFA lesions.

**Methods:**

This is a multicenter retrospective registry of patients with peripheral artery occlusive disease Rutherford categories 2 to 5 treated by surgical profundaplasty (SP) or atherectomy followed by DCB for DFA lesions (symptomatic DFA). The primary endpoint was clinically driven target lesion revascularization (CD-TLR). Overall mortality, target limb reinterventions, major amputation, and major adverse limb events (MALEs) were additionally analyzed.

**Results:**

A total of 373 patients treated for an arteriosclerotic lesion of the DFA between February 2015 and August 2021 were included, 301 treated by SP and 72 with atherectomy and DCB. The rates of chronic limb threatening ischemia (CLTI) were 42.2% and 22.2% (p<0.002) for the surgical and endovascular groups, respectively. A previous DFA intervention was more frequent in the endovascular group (30.6% vs 15.3%; p<0.003). Patients who had an open repair were more likely to have an occlusion of the profunda (34.9% vs 19.7%, p=0.014), severe calcified lesions (26.5% vs 5.6%, p=0.001), and lesions longer than 20 mm (95.7% vs 88.7%, p=0.024). After propensity score matching, no significant differences were found with regard to technical and hemodynamic success. At 24 months, no difference was found in terms of freedom from CD-TLR (95.7% vs 96.8%), freedom from all-cause mortality (94.2% vs 98.5%), freedom from MALE (90.4% vs 93.9%), and amputation-free survival (93.8% vs 97%). Following endovascular therapy, length of stay was significantly lower (p<0.001) and any reintervention on the index limb was more frequent (p=0.039).

**Conclusion:**

Patients with CLTI, occlusion of profunda, severe calcified lesions, and longer lesions are more frequently treated by open surgery, while reinterventions are more commonly treated by atherectomy and DCB. In patients with comparable clinical and lesion characteristics after matching, endovascular and surgical reconstruction of DFA lesions showed similar mid-term clinical outcomes. However, the risk of reintervention at the index limb is higher after endovascular treatment. Randomized studies are now warranted to compare both techniques in terms of medical and financial aspects.

**Clinical Impact:**

Atherectomy followed by DCB of symptomatic DFA is safe and effective. In patients with comparable clinical and lesion characteristics, outcomes are comparable with surgery. However, the risk of reintervention at the index limb is higher after endovascular treatment. Therefore, whenever possible an additional outflow vessel revascularization should be performed by the time of the primary intervention. Randomized studies are warranted to compare endovascular techniques and open surgery also under economic aspects.

## Introduction

Peripheral arterial disease (PAD) is associated with increased morbidity and mortality^1^ and a significantly lower health-related quality of life,^2^ while symptomatic lower limb arteriosclerosis poses an important economic burden from a societal perspective.^3^ Patients with symptomatic PAD often present with lesions of the femoral bifurcation^4^ and femoral endarterectomy with profundaplasty is a common surgical procedure for treatment of intermittent claudication (IC) or chronic limb threatening ischemia (CLTI).^5–7^ Recently, endovascular techniques have been proposed as an alternative to open repair for high-risk patients^8–10^ with acceptable long-term results in single-center cohorts.^9^ However, data from a multicenter registry^10^ showed a quite high reintervention rate of 15.8% with most reinterventions (62%) occurring after plain balloon angioplasty. Atherectomy has been described as a potential endovascular alternative for lesions of the femoral bifurcation.^11^ Debulking prior to drug-coated balloon (DCB) angioplasty might decrease the risk for flow-limiting dissections, remove the calcified part of the plaque, and increase the drug uptake in the vessel wall.^12^

However, there is a paucity of reports regarding the performance of this approach for deep femoral artery disease. Moreover, although published and ongoing trials assess the outcomes of open and endovascular treatment of the common femoral artery (CFA), no head-to-head analysis is available for profunda arteriosclerosis. The aim of this study is to report real-world clinical outcomes in patients with symptomatic PAD who underwent endovascular and surgical repair of profunda lesions.

## Materials and Methods

### Patient Selection

This study is a retrospective evaluation of consecutive patients undergoing open and endovascular DFA reconstruction between February 2015 and August 2021 in 6 European centers.

Inclusion criteria were symptomatic (Rutherford class 2–6) ≥ 70% stenosis of the DFA by angiography with or without occlusion of the superficial femoral artery causing IC or CLTI. Subjects with previous surgical reconstruction of the groin or proximal vessels (aorta, iliac, and CFA) with recurrent or *de novo* stenosis of the DFA could be enrolled. Exclusion criteria were additional outflow vessel revascularization, acute limb ischemia, bypass anastomosis stenosis, and stenosis following the use of a vascular closure device. Patients treated with other endovascular modalities were also excluded.

Regional ethical approval was previously obtained (ethical approval number: 2021-220-f-s). All patients provided written informed consent for the procedure(s) and for the use of their data.

### Device and Procedural Details

The selected treatment option was operator dependent. All lead operators were vascular/endovascular surgeons and/or interventional cardiologists or radiologists. All patients had undergone cross-sectional imaging or duplex ultrasound pre-operatively to plan the endovascular and open procedures. The mode of anesthesia was dependent on local practice, need for concomitant procedures, and patients’ co-morbidities. Devices were used as per manufacturers’ instructions. All patients received clopidogrel 75 mg and aspirin 100 mg for 4 weeks and aspirin (or anticoagulation if indicated) lifelong.

### Clinical Follow-up

Patients were reviewed clinically post-operatively at set intervals as per local departmental practice. They underwent clinically driven imaging based on clinical findings at their postoperative follow-up appointments. Secondary procedures including clinically driven target lesion revascularization (CD-TLR) were performed when patients were found to have new symptoms (eg, new onset claudication, rest pain, or tissue loss) and a stenosis ≥80% within the previously treated limb. Follow-up data were recorded in digital databases.

### Definitions and Outcomes

The main outcome measure was CD-TLR of the DFA. Secondary endpoints were all-cause mortality, vascular reinterventions of the index limb, major amputation, serious adverse events (SAEs), major adverse limb event (MALE), and change in Rutherford class.

Serious adverse events were defined as any clinical fatal event; life-threatening event resulting in persistent or significant disability; need for surgical or percutaneous intervention; or event requiring prolonged hospitalization. MALE was defined as untreated loss of patency, reintervention on the index arterial segment, or amputation of the index limb. We distinguished between reintervention at the level of treated profunda lesion (CD-TLR) and any reintervention at the index limb. The latest pre-operative CT angiography and intra-operative angiogram were used to define the degree and length of disease as well as calcification of the DFA. Calcification of the target lesions was reported using the peripheral artery calcification scoring system (PACSS) classification (grades 0–4: no visible calcification of the target lesion, unilateral wall calcification <5 cm, unilateral calcification ≥5 cm, bilateral wall calcification <5 cm, and bilateral calcification ≥5 cm, respectively).^13^ A grade of 4 was considered as “severe.” A diagnosis of diabetes was recorded based upon past medical case records and pre-existing medications. A diagnosis of hypertension was made based on baseline blood pressure and medication. Chronic kidney disease (CKD) was diagnosed based on latest available estimated glomerular filtration rate (eGFR) level (eGFR <30 ml/min). Acute kidney injury was defined as per the Kidney Disease Improving Global Outcomes criteria.

### Statistical Analysis

Statistical analysis was performed using STATA (Version 16.1; Statistics/Data analysis, StataCorp LLC, College Station, Texas). Continuous variables were presented as mean ± standard deviation if normally distributed and median (with interquartile range [IQR]) if not. Categorical variables were presented in numbers (percentage). Continuous variables were compared using Student’s *t*-test (or Mann-Whitney test if appropriate) and categorical variables were compared using χ^2^ test (or Fisher’s exact test if appropriate).

Propensity score matching (PSM) was performed to compare the same outcomes as described for the entire cohort in an adjusted manner in matched cohorts. Variables included in the PSM process were sex, hypertension, hyperlipidemia, diabetes, coronary artery disease, CKD, previous stroke, previous or current smoking habits, previous treatment of target vessel, CLTI, PACSS score 4, presence of complete occlusion, and lesion length. Propensity score matching was performed in a non-parsimonious fashion with a 1:1 ratio and a caliper of 0.2. Results were presented as odds ratio (OR) with 95% confidence interval (CI).

Time-to-event outcomes were analyzed using Kaplan-Meier curves and life tables. Outcome comparison was performed using log-rank tests and cox proportional hazards model and presented as hazard ratio with 95% CIs. The results were additionally reported at a consistent time point of 24 months.

Sensitivity analysis was performed using 2 methods: multivariable logistic regression and a further logistic regression model using the propensity scores as a linear term. For the multivariable logistic regression, a forward approach for variable selection was used, including variables with p<0.20 on univariate analysis or those considered clinically relevant.

An additional analysis was performed to identify possible predictors for any reintervention at the target vessel with a logistic regression model using a forward approach for variable selection, including variables with p<0.20 on univariate analysis or those considered clinically relevant.

All analyses were considered statistically significant if a 2-tailed p-value of <0.05 was observed.

## Results

### Unmatched Cohort

A total of 373 patients with a DFA lesion (293 male, 78.5%; mean age: 70 ± 10 years) were treated by SP (301; 80.7%) or by atherectomy and DCB angioplasty (72; 19.3%). Demographics and baseline patients’ characteristics are presented in Table 1. Details on devices used are listed in Supplemental Table 1. Directional atherectomy (HawkOne™ catheter in conjunction with the SpiderFX™ Embolic Protection, Medtronic) was used in 61 patients, while rotational atherectomy (Phoenix atherectomy system, Philips) in 11 cases. The most used DCB was the InPact DCB (Medtronic, Minneapolis, Minnesota, USA) in 43 patients. The remaining patients were treated with following DCB: Passeo-18 Lux (Biotronik AG, Bülach, Switzerland), Ranger (Boston Scientific, Marlborough, MA, USA), Stellarex (Philips, Eindhoven, The Netherlands), SeQuent (B. Braun, Melsungen, Germany), and Litos/Tulip (Acotec Scientific, Beijing, China). Distal embolic protection was used in 65 (90.2%) cases.

**Table 1.** Demographics and Baseline Characteristics.

Variable	SP (N=301)	AART (N=72)	p value
Age (median IQR)	70 (65–76)	69 (63–75)	0.59
Male sex (N [%])	241 (80.1)	52 (72.2)	0.14
HTN (N [%])	251 (83.4)	64 (88.9)	0.25
Hyperlipidemia (N [%])	226 (58.8)	49 (68.1)	0.15
DM (N [%])	96 (31.9)	26 (36.1)	0.49
CAD (N [%])	128 (42.5)	40 (55.6)	**0.046**
CKD (N [%])	61 (20.3)	12 (16.7)	0.49
Stroke (N [%])	37 (12.3)	8 (11.1)	0.78
Tobacco use (N [%])	204 (67.8)	45 (62.5)	0.39
Previous treatment of the target vessel (N [%])	46 (15.3)	22 (30.6)	**0.003**
History of inflow procedure (N [%])	24 (8)	10 (13.9)	0.08
CLTI (N [%])	127 (42.2)	16 (22.2)	**0.002**
ABI (N [%])	0.5 (0.34–0.60)	0.5 (0.38–0.64)	0.55
On AAS (N [%])	221 (73.4)	60 (83.3)	0.08
On clopidogrel (N [%])	88 (29.2)	16 (22.2)	0.23
On DOAC (N [%])	37 (12.3)	12 (16.7)	0.32
On statin (N [%])	175 (58.1)	55 (76.4)	**0.004**
On ACE inhibitors (N [%])	176 (58.5)	41 (56.9)	**0.013**

Demographics and baseline variables in both treatment groups in the unmatched cohort. Statistically significant differences are represented in bold.

Abbreviations: AART, atherectomy followed by anti-restenotic therapy; AAS, acetylsalicylic acid; ABI, ankle brachial index; ACE, angiotensin converting enzyme; CAD, coronary artery disease; CKD, chronic kidney disease; CLTI, chronic limb threatening ischemia; DM, diabetes mellitus; DOAC, direct oral anticoagulant; HTN, hypertension; IQR, interquartile range (25th–75th percentile); SP, surgical profundaplasty.

Sixty-eight patients had a previous treatment of the femoral bifurcation which was significantly more frequent in the endovascular group (30.6% vs 15.3%; p<0.003). Indications for treatment were mild/moderate claudication (Rutherford 2—61 patients, 16.3%), severe claudication (Rutherford 3—168 patients, 45%), and CLTI (Rutherford 4, 5, and 6—144 patients, 38.6%). Patients who had an open repair were more likely to have an occlusion of the DFA (34.9% vs 19.7%, p=0.014), severe calcification (26.5% vs 5.6%, p=0.001), and lesions longer than 20 mm (95.7% vs 88.7%, p=0.024) compared to patients treated by atherectomy and DCB angioplasty. Lesion characteristics are listed in Table 2.

**Table 2.** Anatomic Characteristics of Both Cohorts.

Variable	SP (N=301)	AART (N=72)	p value
Occlusion (N [%])	98 (34.9)	14 (19.7)	**0.014**
PACSS score >3 (N [%])	181 (64.9)	34 (47.9)	**0.009**
Lesion length (N [%])	34 (26–51)	30 (20–40)	0.81
Lesion length ≥20 mm (N [%])	267 (95.7)	63 (88.7)	**0.024**

Anatomic baseline differences between groups. Statistically significant differences are represented in bold.

Abbreviations: AART, atherectomy followed by anti-restenotic therapy; PACSS, peripheral artery calcification scoring system; SP, surgical profundaplasty.

#### Thirty-day outcomes

Table 3 summarizes the 30-day outcomes of the unmatched cohort. Four patients died (3 after surgery) and 3 required a major lower limb amputation (2 after surgery). Patients treated by surgery stayed on average 3 days more in hospital (6 vs 3 days after atherectomy; p<0.001) and suffered more frequently a SAE (19.7% vs 6.9%; p=0.014). In both cohorts, ankle brachial index (ABI) significantly improved in the immediate postoperative period (p=0.013).

**Table 3.** Thirty-Day Outcomes.

Variable	SP (N=301, N [%])	AART (N=72, N [%])	OR (95% CI) for categorical outcomes and coefficient (95% CI) for continuous outcomes, p value (univariable)
Technical failure	0 (0)	1 (1.4)	**NA**
Length of stay (days)	6 (5–8)	3 (2–3)	**Coefficient = −2.66 (−3.58, −1.73), <0.001**
ABI post-operatively	0.85 (0.7–1.0)	0.79 (0.63–1.0)	**Coefficient = −0.01 (−0.01, 0.05)**, 0.73
Increase in ABI	0.44 (0.30–0.65)	0.21 (0.10–0.43)	**Coefficient = −0.09 (−0.16, −0.2), <0.013**
In-hospital SAE	59 (19.7)	5 (6.9)	**0.30 (0.12–0.79), 0.014**
30-day mortality	3 (1)	1 (1.4)	1.39 (0.14**–**13.6), 0.29
30-day amputation	2 (0.7)	1 (1.4)	2.11 (0.19**–**23.55), 0.55

Thirty-day outcomes of the unmatched cohort. Statistically significant outcomes are represented in bold.

Abbreviations: AART, atherectomy followed by anti-restenotic therapy; ABI, ankle brachial index; CI, confidence interval; OR, odds ratio; SAE, serious adverse event; SP, surgical profundaplasty.

#### Mid-term outcomes

The median follow-up was 40 months (IQR: 26–50 months) for the surgical group and 29 months (IQR: 17–48 months) for patients treated by debulking and DCB. No patient was lost to follow-up. Twenty-four patients (8.1%) of the open group and 2 patients of the endovascular group (2.8%) died (Table 4). Major amputation, CD-TLR, and MALE did not differ significantly between the 2 groups and were, respectively, 1.0%, 5.4%, 5.8% (SP) and 1.4%, 6.9%, 8.3% (atherectomy and DCB). Vascular reinterventions of the index limb were significantly more frequent in the endovascular group (22.2% vs 8.5%). The observed decrease in Rutherford class was statistically significant for both treatment modalities (p<0.001) without difference between the groups.

**Table 4.** Mid-term Outcomes.

Variable	SP (N=301, N [%])	AART (N=72, N [%])	OR (95% CI) for categorical outcomes and coefficient (95% CI) for continuous outcomes, p value (univariable)
Decrease in Rutherford class	2 (2–3)	2 (0–2)	**Coefficient = −0.72 (−1.07, −0.38), <0.001**
Any reintervention	25 (8.5)	16 (22.2)	**3.07 (1.54−6.13), 0.001**
CD-TLR	16 (5.4)	5 (6.9)	1.30 (0.46**−**3.66), 0.62
Major amputation	3 (1.0)	1 (1.4)	1.37 (0.14–13.33), 0.79
Overall mortality	24 (8.1)	2 (2.8)	0.32 (0.07**−**1.39), 0.13
MALE	17 (5.8)	6 (8.3)	1.47 (0.56**−**3.89), 0.43
Death or MALE	39 (13.3)	8 (11.1)	0.82 (0.36**−**1.83), 0.62
Death or major amputation	26 (8.8)	3 (4.2)	0.45 (0.13**−**1.53), 0.20

Mid-term outcomes of the unmatched cohort. Statistically significant outcomes are represented in bold.

Abbreviations: AART, atherectomy followed by anti-restenotic therapy; CD-TLR, clinically driven target lesion revascularization; CI, confidence interval; MALE, major adverse limb event; OR, odds ratio; SP, surgical profundaplasty.

**Figure 1.**
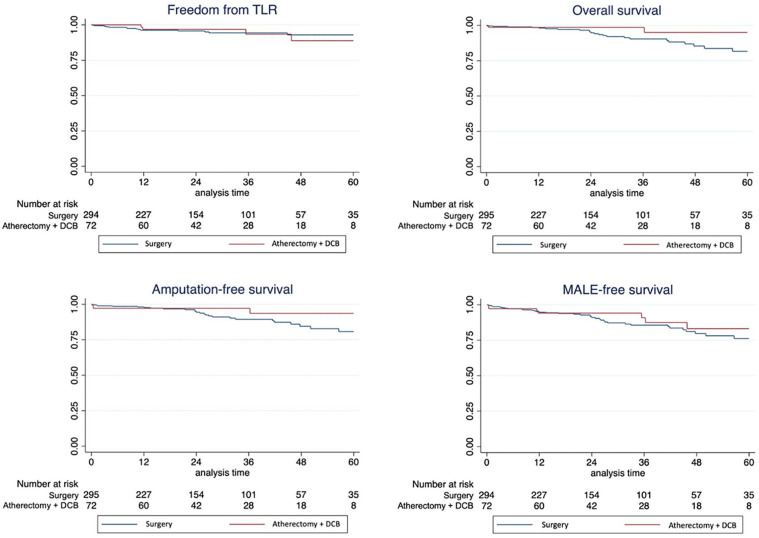
Kaplan-Meier plots showing no significant differences in freedom from clinically driven target lesion revascularization (CD-TLR), overall survival, amputation-free survival, and major adverse limb event (MALE)-free survival between endovascular and surgical reconstruction of the deep femoral artery. Abbreviation: DCB, drug-coated balloon.

No significant differences were found in freedom from CD-TLR, overall survival, amputation-free survival, and MALE-free survival. The corresponding Kaplan-Meier plots and data analysis are shown in Figure 1.

### Propensity Score–Matched Cohorts

To compare outcomes in an adjusted manner, PSM was performed. Baseline characteristics were well balanced between groups (Table 5) and results are presented in Table 6.

**Table 5.** Demographics and Baseline Characteristics of the Matched Cohort.

Variable	SP (N=69)	AART (N=69)	p value
Age (median IQR)	71 (65–75)	70 (63–76)	0.76
Male sex (N [%])	52 (75.4)	50 (72.5)	0.70
HTN (N [%])	63 (91.3)	61 (88.4)	0.57
Hyperlipidemia (N [%])	42 (60.9)	46 (66.7)	0.48
DM (N [%])	25 (36.2)	24 (34.8)	0.86
CAD (N [%])	39 (56.5)	38 (55.1)	0.86
CKD (N [%])	12 (17.4)	12 (17.4)	>0.99
Stroke (N [%])	6 (8.7)	7 (10.1)	0.77
Tobacco use (N [%])	46 (66.7)	44 (63.8)	0.72
Previous treatment of the target vessel (N [%])	18 (26.1)	20 (29.0)	0.70
CLTI (N [%])	17 (24.6)	16 (23.2)	0.84
On AAS (N [%])	53 (76.8)	58 (84.1)	0.28
On clopidogrel (N [%])	22 (31.9)	15 (21.7)	0.18
On DOAC (N [%])	10 (14.5)	12 (17.4)	0.64
On statin (N [%])	43 (62.3)	53 (76.8)	0.06
On ACE inhibitors (%)	42 (60.9)	39 (56.5)	0.60
Occlusion (N [%])	15 (21.7)	14 (20.3)	0.83
PACSS score >3 (%)	31 (44.9)	34 (49.3)	0.61
ABI	0.5 (0.4–0.6)	0.5 (0.38–0.62)	0.89
Lesion length (N [%])	30 (23–36)	30 (20–40)	0.61

Propensity score–matched cohort—demographics and baseline differences between treatment groups.

Abbreviations: AART, atherectomy followed by anti-restenotic therapy; AAS, acetylsalicylic acid; ABI, ankle brachial index; ACE, angiotensin converting enzyme; CAD, coronary artery disease; CKD, chronic kidney disease; CLTI, chronic limb threatening ischemia; DM, diabetes mellitus; DOAC, direct oral anticoagulant; HTN, hypertension; IQR, interquartile range (25th–75th percentile); PACSS, peripheral artery calcification scoring system; SP, surgical profundaplasty.

**Table 6.** Outcomes of the Propensity Score–Matched Cohort.

Variable	SP (N=69, N [%])	AART (N=69, N [%])	OR (95% CI) for categorical and coefficient (95% CI) for continuous outcomes, p value
Technical failure	0 (0)	1 (1.4)	NA
Length of stay (days)	5 (4–6)	3 (2–3)	**Coefficient = −2 (−3, −1), <0.001**
ABI post-operatively	0.80 (0.70–1.0)	0.78 (0.66–0.90)	Coefficient = **−**0.01 (**−**0.1, 0.09), 0.86
Increase in ABI	0.40 (0.16–0.50)	0.22 (0.10–0.40)	Coefficient = **−**0.05 (**−**0.14, **−**0.04), 0.28
In-hospital SAE	9 (13.0)	5 (7.2)	0.52 (0.16–1.64), 0.27
30-day mortality	0 (0)	1 (1.4)	NA
30-day amputation	0 (0)	1 (1.4)	NA
Decrease in Rutherford class	2 (1–2)	2 (0–2)	Coefficient = **−**0.29 (**−**0.76, 0.17), 0.21
Any reintervention	6 (8.7)	15 (21.7)	**2.92 (1.06–8.04), 0.039**
CD-TLR	2 (2.9)	4 (5.8)	2.06 (0.36–11.64), 0.41
Major amputation	0 (0)	1 (1.4)	NA
Overall mortality	4 (5.8)	2 (2.9)	0.48 (0.09–2.74), 0.41
MALE	2 (2.9)	5 (7.2)	2.62 (0.49–13.98), 0.26
Death or MALE	6 (8.7)	7 (10.1)	1.18 (0.38–3.72), 0.77
Death or major amputation	4 (5.8)	3 (4.3)	0.74 (0.16–3.43), 0.70

Treatment outcomes in the propensity score–matched cohort. Statistically significant outcomes are represented in bold.

Abbreviations: AART, atherectomy followed by anti-restenotic therapy; ABI, ankle brachial index; CD-TLR, clinically driven target lesion revascularization; CI, confidence interval; MALE, major adverse limb event; OR, odds ratio; SAE, serious adverse event; SP, surgical profundaplasty.

#### Thirty-day outcomes

No differences were observed regarding technical failure and hemodynamic success. Postoperative ABI was 0.80 and 0.78 in the surgical and endovascular group, respectively. The increase in ABI was not statistically significant between the endovascular and the surgical group. Serious adverse event, 30-day mortality, and 30-day major amputation were also not different. However, in the endovascular group length of stay was significantly lower (Coefficient: −2 days [95% CI: −3, −1], p<0.001).

#### Mid-term outcomes

At 24 months, no difference was found in terms of freedom from CD-TLR (95.7% vs 96.8%), freedom from all-cause mortality (94.2% vs 98.5%), freedom from MALE (90.4% vs 93.9%), and amputation-free survival (93.8% vs 97%) between the surgical and endovascular groups, respectively. However, reinterventions on the index limb were more frequent in the endovascular group (OR: 2.92 [95% CI: 1.06–8.04]).

### Sensitivity Analysis

Sensitivity analysis was performed using 2 methods: multivariable logistic regression (Supplemental Table 2) and a further logistic regression model using the propensity scores as a linear term (Supplemental Table 3). Both analyses showed similar results to the ones found on the propensity score–matched analysis detailed above.

### Additional Analysis

A reintervention was performed in 41 (11%) patients. To identify predictors of reinterventions on target limb, we performed an additional logistic regression model (Supplemental Table 4). The use of atherectomy followed by DCB (OR: 3.88) was the only predictor of reintervention at the affected limb.

## Discussion

Although surgery remains the gold standard for arteriosclerotic lesions of the groin arteries, endovascular therapy has been suggested as an alternative to open repair for CFA disease.^14^ However, little is known regarding the performance of minimally invasive modalities for DFA arteriosclerosis. This multicenter study showed that atherectomy combined with DCB angioplasty and surgical treatment has comparable satisfactory mid-term clinical outcomes and CD-TLR rates in comparable patient cohorts. Therefore, this minimal invasive therapy can be offered to patients who are not fit for surgery (see Table 7). In this registry, the use of the endovascular modality was preferred in patients with restenotic disease, while surgery was more frequently performed in patients with occlusive longer and more calcified lesions as well as in patients with CLTI.

**Table 7.** Factors That Favor Atherectomy Combined With DCB Angioplasty Versus Open Surgery for Treatment of Profunda Artery Lesions.

*Factors that favor atherectomy and DCB* Extreme obesity Scar in the groin Previous groin procedure Limited life expectancy Comorbidities (ie, severe COPD, angina, cardiac failure) Candidate for TAVI *Factors that favor open surgery* Severe calcified lesion Long lesion Total occlusion Potential atheromatous embolism Severe ischemic symptoms

Abbreviations: COPD, chronic obstructive pulmonary disease; DCB, drug-coated balloon; TAVI, transcatheter aortic valve implantation.

Recent and ongoing research aimed to evaluate the safety and efficacy of different endovascular modalities for arteriosclerotic lesions of the groin vessels. The Traitement des Lésions Athéromateuses de l’Artère Fémorale Commune par Technique Endovasculaire Versus Chirurgie Ouverte study was the first randomized, multicenter trial that evaluated the performance of primary stenting and surgical repair for *de novo* lesions of the CFA.^14^ Similar to our findings, no difference was observed regarding the clinical performance and the reintervention rates of the 2 groups at 2 years.

After the early descriptions of plain angioplasty as therapeutic option of profunda lesions,^15^ this approach has been primarily used in patients with scars in the groin or compromised lymph with potential increased risk of wound infection.^16^ In our cohort, patients treated by endovascular therapy have more frequently undergone a previous reconstruction of the DFA. To date, results from single-center series have demonstrated acceptable results of balloon angioplasty.^8,9,16^ However, consecutive patient series and systematic review of the literature showed low cumulative patency and reintervention-free survival rates at 3 years.^17^ Angioplasty with bail-out stenting improved the outcomes of endovascular treatment in single-center cohorts.^9,16^ But dedicated stents for this indication and large, multicenter evaluations are not available. In addition, the lesion often involves the profunda take-off and stenting of the ostium would potentially obstruct the femoral bifurcation. Even if the hinge point of the hip bending zone is more proximal, there is a reluctance to perform primary stenting in the groin arteries.^18^ In a large multicenter registry,^12^ only 18.2% of the patients were treated with stents and stent fractures are not rare.^16^

The introduction of DCBs led to a paradigm shift in the treatment of peripheral occlusive disease and vessel preparation improves the results of DCBs.^19^ This combination extended the use of “leave nothing behind” strategies in so-called “no stenting zones.”^20^ A subgroup analysis of the CONFIRM registries^21^ demonstrated that orbital endarterectomy is feasible and safe for the treatment of calcific profunda lesions. However, long-term outcome after orbital atherectomy of the profunda artery is unknown and a comparison with SP has not be performed so far. In addition, orbital atherectomy recently demonstrated inferior luminal gain compared to directional atherectomy using intravascular imaging.^22^ In our study, atherectomy followed by DCB was associated with satisfactory freedom from CD-TLR after a mean follow-up of 29 months. Overall mortality, amputation-free survival, and MALE-free survival were comparable with a matched surgical cohort. Also, the risk of distal embolization which is intrinsic during debulking of calcified lesions was minimized by using filters in 90.2% of the cases.

Of note, any reintervention rate at index limb but not CD-TLR at the DFA was significantly higher in the endovascular group (22.2% vs 8.5%). The data suggest that, whenever possible, an additional outflow vessel revascularization should be performed at the primary intervention in some patients. In this context, isolated profunda endovascular revascularization remains controversial as primary approach especially in CLTI patients.^23^ In our study, 11% of the patients received a reintervention at the index limb, but only 15 (4%) required bypass surgery during the follow-up. Noteworthy, CLTI was not found to influence any multivariate model. Probably, the majority of CLTI patients included in this study showed an adequate collateral pathway from the profunda to the below-the-knee arteries. As recommended by other authors,^5–7,16,18^ the operators involved in the study consider crucial this anatomic criterion especially in CLTI patients which explains the relatively low reintervention rate in our cohort.

### Limitations

There are some limitations to this study that are inherent to all registries. The design of the study was non-randomized, and no provision was made for a core lab. In addition, the small number of comparable cases in the endovascular group may limit the power to detect a difference in CD-TLR. The primary endpoint of the study was CD-TLR which could include operator or physician bias. As peak systolic velocity was not evaluated at all predetermined time points, we were not able to calculate primary and secondary patency. Despite exact evaluation of the target lesions, additional information about the quality of the run-off was not recorded. The results represent the current practice in 7 experienced institutions and may not represent all vascular centers. Finally, the results need to be confirmed in prospective confirmatory studies.

## Conclusion

Drug-coated balloon angioplasty following atherectomy of the profunda artery is safe and effective in the treatment of symptomatic PAD. Also, at mid-term follow-up the outcomes are comparable with the SP so that the endovascular approach appears promising in mainly restenotic disease of the profunda artery. However, reintervention rate on the index limb but not at the index lesion is higher in the endovascular group. Long-term evaluation and prospective studies involving more institutions are required.

## Supplemental Material

sj-docx-1-jet-10.1177_15266028241284443 – Supplemental material for Atherectomy Followed by Drug-Coated Balloon Angioplasty Versus Surgery for Symptomatic Deep Femoral Artery Arteriosclerotic Disease Supplemental material, sj-docx-1-jet-10.1177_15266028241284443 for Atherectomy Followed by Drug-Coated Balloon Angioplasty Versus Surgery for Symptomatic Deep Femoral Artery Arteriosclerotic Disease by Giovanni Battista Torsello, Ryan Gouveia e Melo, Thomas Zeller, Tanja Böhme, Grigorios Korosoglou, Raphael Coscas, Konstantinos Stavroulakis, Dimitrios Kapetanios, Giovanni Federico Torsello and Bahaa Nasr in Journal of Endovascular Therapy
