# Supplementary material for: Atherectomy Followed by Drug-Coated Balloon Angioplasty Versus Surgery for Symptomatic Deep Femoral Artery Arteriosclerotic Disease
Source: J Endovasc Ther. 2024 Oct 18;33(3):1186–94. doi: 10.1177/15266028241284443 (PMC13172112; doi:10.1177/15266028241284443)
Supplement: sj-docx-1-jet-10.1177_15266028241284443 – Supplemental material for Atherectomy Followed by Drug-Coated Balloon Angioplasty Versus Surgery for Symptomatic Deep Femoral Artery Arteriosclerotic Disease [file sj-docx-1-jet-10.1177_15266028241284443.docx]

**Supplementary Table 1** Endovascular devices and patch material used.

*Atherectomy*

Directional 61

Rotational 11

Total 72

*Patch material*

Synthetic 224

Biologic 65

Autologous 12

Total 301

**Supplementary Table 2** Treatment outcomes in the logistic regression model using a forward approach.

| Variable | Adjusted OR/Coef (95%C) and p value |
| --- | --- |
| In hospital SAE | 0.33 (0.11-1.02), 0.05 |
| 30-day mortality | 0.99 (0.024-40.40), 0.99 |
| 30-day amputation | 1.99 (0.14-28.50), 0.61 |
| Any re-intervention | 3.28 (1.34-8.01), 0.009 |
| CD-TLR | 1.05 (0.30-3.73), 0.93 |
| Major amputation | 1.96 (0.07-56.54), 0.69 |
| Overall mortality | 0.41 (0.06-2.61), 0.34 |
| MALE | 1.28 (0.40-4.12), 0.68 |
| Periop death or MALE | 0.79 (0.29-2.13), 0.64 |
| Death or major amputation | 0.53 (0.11; 2.57), 0.43 |

In bold are statistically significant outcomes. Abbreviations - SAE: serious adverse event. CD-TLR: clinically driven target lesion revascularization; MALE: major adverse limb event.

**Supplementary Table 3** Treatment outcomes in the logistic regression model using a the propensity scores as a linear term.

| Variable | Propensity score analysis OR (95%CI), p value |
| --- | --- |
| In hospital SAE (n=343) | 0.46 (0.07-3.03), 0.43 |
| 30-day mortality (n=343) | 5.58 (0.03-1026.74), 0.52 |
| 30-day amputation (n=344) | 0.41 (0.00-1125.10), 0.82 |
| **Any re-intervention (n=338)** | **7.06 (1.12-44.57), 0.038** |
| CD-TLR (n=338) | 1.15 (0.07-18.02), 0.92 |
| Major amputation (n=338) | 0.00 (0.00-239.96), 0.18 |
| Overall mortality (n=339) | 0.16 (0.01-3.54), 0.24 |
| MALE (n=337) | 0.76 (0.05-11.79), 0.84 |
| Periop death or MALE (n=338) | 0.35 (0.04-3.02), 0.34 |
| Death or major amputation (n=339) | 0.09 (0.00; 2.03), 0.13 |

In bold are statistically significant outcomes. Abbreviations - SAE: serious adverse event; CD-TLR: clinically driven target lesion revascularization; MALE: major adverse limb event.

**Supplementary Table 4** Additional analysis to identify predictors of index limb reinterventions using a logistic regression model.

| Variable | Adjusted OR (95%C) and p value |
| --- | --- |
| Atherectomy (versus surgery) | 3.88 (1.55-9.72), 0.004 |
| DM | 2.21 (0.93-5.26), 0.07 |
| ABI | 0.19 (0.02-1.54), 0.12 |
| On ACE inhibitors | 2.07 (0.79-5.39), 0.14 |
| Occlusion (versus stenosis) | 0.73 (0.26-2.08), 0.56 |
| Lesion length (mm) | 0.98 (0.95-1.01), 0.28 |
| Age (years) | 0.98 (0.95-1.01), 0.19 |
| PACCS ≥3 | 1.03 (0.42-2.52), 0.95 |
| CLTI (versus claudicant) | 0.91 (0.31-2.69), 0.87 |

In bold are statistically significant outcomes. Abbreviations - DM: diabetes mellitus; PACCS: Peripheral Arterial Calcium Scoring Scale; CLTI: chronic limb threatening ischemia.
